# Behind the Counter

**Published:** 1893-05

**Authors:** 


### BEHIND THE COUNTER.

No. 5.

On entering the store the Monday following my last communication, I was directed to report to the head of stock of the hosiery department. Using the floor-walker as my guide, I was soon brought face to face with this important personage. She proved to be a person of most agreeable manners, in the very prime of womanhood, who received me so kindly that I was put immediately at my ease. "You have been so favorably spoken of by the manager of the kitchen department that I am confident we shall get along very well together," she remarked in a quiet patronizing way, as she waved me to a place in front of the neatly arranged stock which was thenceforth to command my attention, under her direction, and I busied myself in making its near acquaintance preparatory to answering the multifarious demands of customers, as to material, size and price of these nether appendages in general, and thus the day dragged out its weary length with scarcely an incident to relieve its commonplace monotony. I confess to a feeling of homesickness for my pots and kettles and the cheery companionship of 13, nor could I refrain from looking upon my promotion as a kind of banishment, more especially as a quite necessary change in my lunching time, forbade our contemplated noon-day tête-à-tête to our mutual disappointment, but 13 was able to accommodate hers to mine after a short interval, so that now we seldom miss spending the customary half hour together and whilst our lunch baskets are being depleted, we are quite sure to enliven the frugal repast with intellectual dainties no less relishable.

I must not omit to speak of my visit to my friend in her snug little home in that portion of the city, which only a decade ago was given over to whole communities of squatters, whose fantastic habitations made up of odd sections of accommodating patchwork, with a liberal allowance of scrawny quadrupeds and straggling flocks of geese, covered all the available space and gave the neighborhood an air of precarious holdings and unstable proprietorship.

Now, all this is changed as by the waving of a magic wand, and the west side as it is called, is the seat of new and handsome private residences, bordering upon noble streets and avenues, swarming with busy life of the great and growing metropolis.

It was here that the elevated train delivered us, and after a walk of less than two blocks, we entered an imposing edifice, whose interior indicated that it was designed for occupancy by a number of distinct families. As we approached the upper landing, a lad, tall, slim, and becomingly attired, sprang forward and embracing my friend, remarked "Oh, mother, how well I know your step." It was not until after the indulgence of mutual caresses that Arthur was duly presented. "I knew you were coming," he said, still holding my hand, "and I was ever so glad, for we are so much by ourselves it will be a relief to mamma to have you with us if only for a few hours." At this I caught him in my arms with a real motherly hug, assured that his good qualities had not been overdrawn by an over indulgent mother. Before laying aside my wraps I indulged in a hasty outlook from the windows. The view was magnificent. To the east, stretching far and wide lay the park with its graceful undulations, and promise of an early "wearing of the green," whilst to the westward, far over a very wilderness of house roofs flowed the majestic Hudson, and gave back the tender coloring of sunset ; and further on, the forest crowned hills of Jersey, dotted here and there with picturesque villas that denoted the comforts and refinements of home, sweet home !

"Lay off your things in any convenient place, Lina, and make yourself perfectly at home, in a few minutes we shall ask you to join us at the tea table," said Mildred (for so she requested me to call her), on withdrawing to the rear of her premises, whence I followed to lend a hand in case my services could be made available.

The cozy tea table, with its abundance and good cheer was in harmony with all the rest, and ere we finished our repast, Arthur and I had added a double link to the golden chain that bound its dual inmates in fraternal love.

The passing hours glided so smoothly away that the time of parting came all too soon when both mother and son insisted upon attending me to the cars that were to take me to my lodgings. It is such companionship and such a home as theirs that more than recompense the adversities that beset our lives and fill the weariest of our days with gladness.

Now, indeed, could I understand how it was that Mildred, even in her darkest hours, was sustained by a great hope that was ever present to her inner sense, and like a gleaming star shone above the barriers that hedged in her life.

We said good night, a lingering hesitating good night, with mutual regrets and promises, as the ample vehicle rolled away on its iron track, leaving me to my own little world of fancies and meditations. Kinder friends I shall never know. They have even urged me to bring Walter with me on my very next visit. Perhaps you opine I will not do so!

PAULINE.

